# Efficacy of Spectral-Aided Visual Enhancer in Classification of Esophageal Cancer

**DOI:** 10.3390/cancers18101609

**Published:** 2026-05-15

**Authors:** Kok-Yean Koh, Arvind Mukundan, Riya Karmakar, Chaudhary Tirth Atulbhai, Tsung-Hsien Chen, Wei-Chun Weng, Hsiang-Chen Wang

**Affiliations:** 1 Division of Gastroenterology and Hepatology, Department of Internal Medicine, Ditmanson Medical Foundation Chia-Yi Christian Hospital, Chia-Yi 60002, Taiwan; yunxinkoh2@yahoo.com.tw; 2 Department of Mechanical Engineering, National Chung Cheng University, 168, University Rd., Min Hsiung, Chia-Yi 62102, Taiwan; arvindmukund96@gmail.com (A.M.); karmakarriya345@gmail.com (R.K.); 3 Department of Biomedical Imaging, Chennai Institute of Technology, Sarathy Nagar, Chennai 600069, India; 4 Department of Computer Science Engineering, School of Engineering and Technology, Sanjivani University, Singnapur 423603, India; 5 Department of Integrated Bachelor of Technology, School of Engineering and Technology, Sanjivani University, Singnapur 423603, India; 6 Department of Mechanical and Industrial Engineering, Indian Institute of Technology Roorkee, Roorkee—Haridwar Highway, Roorkee 247667, India; t_chaudhary@me.iitr.ac.in; 7 Department of Internal Medicine, Ditmanson Medical Foundation Chia-Yi Christian Hospital, Chia-Yi 60002, Taiwan; cych13794@gmail.com; 8 Department of Gastroenterology, Kaohsiung Armed Forces General Hospital, 2, Zhongzheng 1st. Rd., Lingya District, Kaohsiung City 80284, Taiwan; 9 Department of Medical Research, Dalin Tzu Chi Hospital, Buddhist Tzu Chi Medical Foundation, No. 2, Minsheng Road, Dalin, Chia-Yi 62247, Taiwan; 10 Hitspectra Intelligent Technology Co., Ltd., Kaohsiung 80661, Taiwan

**Keywords:** esophageal cancer, machine learning, SAVE, artificial intelligence, narrow-band imaging, YOLO, white-light imaging

## Abstract

**Background/Objectives:** Esophageal cancer is one of the major global causes of cancer mortality, and the 5-year survival rate remains below 20% because many cases are detected late. In this study, a Spectral-Aided Vision Enhancer (SAVE) algorithm was utilized to convert conventional white-light endoscopic images (WLI) into hyperspectral-like narrow-band imaging (NBI) images for machine-learning classification of Dysplasia, Normal, and Squamous Cell Carcinoma (SCC). **Methods:** A total of 762 WLI images obtained from Kaohsiung Medical University were augmented to 1074 using the Al bumentations library, employing vertical flipping, horizontal flipping, and rotations. The SAVE conversion pipeline employs a 24-patch Macbeth color checker for calibration, γ-correction, CIE XYZ transformation, and multivariate regression to interpolate spectral bands, yielding an average color difference of 2.79 (CIEDE2000) from true NBI. The training outcomes and performance metrics illustrate the versatility of the machine learning/deep learning models—Random Forest (RF), Support Vector Machine (SVM), and Convolutional Neural Network (CNN)—which were trained and evaluated on both the original WLI and SAVE datasets. Performance metrics were analyzed based on precision, recall, accuracy, and F1-score. **Results:** The CNN sample achieved an accuracy of 100 percent on SAVE data, compared to 93 percent for WLI. The accuracy of RF improved, with WLI at 91% and SAVE at 96%, while SVM increased from 79% to 84%. These improvements indicate the diagnostically valuable spectral variations that can be amplified with SAVE, resulting in significant enhancements in pre-cancer/SCC sensitivity. **Conclusions:** The proposed SAVE method demonstrates significant potential for enhancing endoscopic imaging and advancing computer-aided diagnosis in esophageal cancer screening, with applicability in other gastrointestinal imaging scenarios as well.

## Simple Summary

Esophageal cancer (EC) has a high mortality rate and needs early detection to improve patient survival. This study used SAVE technology to convert standard white-light imaging (WLI) images into SAVE images with enhanced spectral information. Random Forest, CNN, and SVM models were then used to evaluate both WLI and SAVE images. The results showed that SAVE images performed better than WLIs in classifying different EC categories. All models improved in accuracy, with at least a 3% increase when using SAVE images. SAVE images also showed better performance in other evaluation metrics, including precision, recall, and F1 score. These findings suggest that SAVE may improve image-based classification performance and could potentially support computer-aided assessment of esophageal lesions. However, prospective clinical studies with biopsy confirmation, patient-level validation, and outcome analysis are required before determining its impact on clinical decision-making, mortality, or healthcare costs.

## 1. Introduction

Esophageal cancer (EC) is the sixth leading cause of cancer-related mortality globally and ranks ninth among the most prevalent cancers [1]. In 2018 alone, more than 572,000 people were diagnosed with EC [2], which is responsible for around 450,000 deaths every year [3]. The rate of 5-year survival for EC, which is below 20% for diagnosed patients [4], underscores the critical need for early detection and treatment. EC incidence and fatality rates are 2–3 times greater in males than females [5]. Less developed regions carry the most significant burden of this malignant tumor, with around 80% of all cases occurring there [6]. ECs can be classified into two main categories: esophageal adenocarcinoma (EAC), which is prevalent in North America and Western Europe, and esophageal squamous cell carcinoma (ESCC), which is widespread in Eastern Europe and Asia [7]. ESCC has the largest share among all ECs regarding the incidence of cancer, accounting for around 85% of total EC cases [8].

The precursor of ESCC is esophageal squamous dysplasia (ESD). It is an asymptomatic precursor lesion and the degree of dysplasia varies for the subsequent risk of cancer [9]. Neoplastic changes to the esophageal squamous epithelium without invasion are known as squamous dysplasia [10]. Dysplasia was traditionally divided into three grades: mild, moderate, and severe, with each increase in grade increasing the chance of progression to ESCC [11]. In the US, the incidence of EAC is around five times greater in white males than in black males, while for ESCC, the incidence is three to four times higher in black males than in white males [12]. About 40% of ESCC cases in the US were classified as metastatic and incurable and 32% involved lymph nodes and local organs. At five years, the overall survival rate is less than 30% [13].

Recent technological advances have allowed earlier cancer detection using traditional RGB images combined with machine learning (ML), which has shown promising results [14]. For instance, de Groof et al. [15] developed a hybrid ResNetUNet model, which attained an accuracy of 88%, sensitivity of 93%, and specificity of 83%; similarly, Zhang et al. [16] developed a two-stage DLS model with two selection and classification networks for detecting EC, which achieved 90.3%, 92.5%, and 88.7% accuracy, sensitivity, and specificity, respectively. Chen et al. [17] made a faster R-CNN EC detection model utilizing 1520 gastrointestinal CT images from around 420 patients. They reached an F1 score of 95.71% and mAP reached 92.15% with an average detection time of 5.3 s per CT image. Tang et al. [18], proposed a multi-task classification and segmentation consisting of ELSNet and ELCNet for classification and segmentation tasks, respectively, based on the VGG-16 model. The dataset consisted of 1003 esophageal images from 319 patients and achieved an accuracy of 93.43% along with 92.82% sensitivity and 96.20% specificity.

While traditional white-light imaging (WLI) images provide images based on only three colors: red, blue, and green [19], hyperspectral imaging (HSI) offers a more comprehensive approach. HSI can capture many closely spaced spectral bands, ranging from visible to infrared regions of the electromagnetic spectrum [20]. Each object exhibits a unique reflectance so that HSI can separate different objects from each other [21]. HSI is used regularly for research in various fields, such as remote sensing [22], geology [23], astronomy [24], agriculture [25], defense [26], archeology [27], and many more. This innovative technology holds great potential for enhancing our understanding of cancer and improving early detection methods, sparking curiosity and interest among researchers and professionals.

Narrow-band imaging (NBI) is an endoscopic imaging technique that supposedly enhances the visualization of the mucosa’s vasculature network and surface texture to enable improved tissue characterization, diagnosis, and differentiation [28]. NBI uses a shorter wavelength of visible light, which peaks at 415 nm and 540 nm. Its low tissue permeability makes it ideal for observing mucosal surface structures [29]. Gai et al. [30] evaluated the efficacy of NBI in detecting early EC and found that NBI was superior to ordinary endoscopy, i.e., WLI. Similarly, Ye et al. [31], conducted a study comparing NBI’s and WLI’s efficacy in detecting non-invasive bladder cancer. They found that NBI had sensitivity, specificity, and false favorable rates of 97.70%, 50%, and 50%, respectively, while WLI had 66.67%, 25%, and 75% for the same aspects, respectively.

Therefore in this study, a novel HSI conversion algorithm capable of converting a WLI into a NBI image through spectral reconstruction has been developed and has been evaluated on the esophageal cancer classification to detect dysplasia and SCC based on indicators such as precision, F1 score, recall, and accuracy comparing the WLI and the corresponding SAVE dataset through machine learning models such as RF, SVM, and CNN models.

## 2. Materials and Methods

### 2.1. Dataset

The data utilized in this paper was obtained from the Ditmanson Medical Foundation Chia-Yi Christian Hospital utilizing a CV-290 Olympus (Olympus Corporation, Tokyo, Japan), which is a conventional endoscope. The study utilized endoscopic pictures from 150 patients, comprising 50 normal cases, 50 dysplastic cases, and 50 cases of SCC. The patients were aged between 40 and 70 years, with a male-to-female ratio of approximately 7:3. Patient’s images were included if they possessed high-quality WLI images for analysis and were classified into one of three diagnostic categories: normal, dysplasia, or SCC. Images with inferior quality, significant artifacts, inadequate visualization of the mucosal region, and incomplete clinical information were removed. The primary objective of this study was image-based categorization utilizing WLI and SAVE-transformed pictures; hence, no comprehensive subgroup analysis based on illness stage was performed. To mitigate the danger of data leaking, the dataset was partitioned at the patient level rather than at the image level. Consequently, the training, validation, and testing datasets did not contain images from the same patient. Data augmentation was implemented solely after the datasets were divided and was restricted to the training set; no augmented versions of testing images were included in the training data. The independent test set was only utilized in the final model assessment. A total of 762 WLIs in the dataset are sourced from the Ditmanson Medical Foundation Chia-Yi Christian Hospital in .jpg format. The dataset is divided into three classes: normal, dysplasia, and SCC as shown in Table 1. Dysplasia is the precursor lesion to SCC, so more priority is given to detecting it. However, it often looks similar to SCC, so differentiating it becomes challenging. Out of 762 images, normal class was assigned to 243 images, SCC to 241, and dysplasia to 278. Data augmentation was also used on some of these images, i.e., 93 images of normal class, 128 of dysplasia, and 91 of SCC class. The Albumentations library of Python was used for augmentation. The methods used were HorizontalFlip, VerticalFlip, RandomRotate90—which rotates random images by 90 degrees—and Rotate—which rotates the photos by a maximum of 45 degrees clockwise or anticlockwise. After augmentation, the final dataset had 336 images in the normal class, 406 in dysplasia, and 332 in SCC. With this, the total size of the dataset reached 1074 images after augmentation.

The final dataset was then converted to SAVE images utilizing the SAVE technique, increasing the intensity and contrast of blue and green colors. While importing the dataset in the models, all images were either cropped to or maintained a maximum pixel dimension of 640 × 640. This new dataset was then compared with the original WLIs by randomly splitting the datasets into 85% for training the models, 5% for validation and 10% for testing the model in the CNN. For the other models, the dataset was partitioned into two groups: 85% for training the model and 15% for validating the model. The primary data consisted of authentic WLIs of patients. The WLIs were not turned into a separate real endoscopic dataset; instead, they were computationally transformed into SAVE images via the proposed spectral reconstruction pipeline. The SAVE images are augmented representations of NBI images derived from actual WLI endoscopic images, rather than being directly obtained from hardware-based HSI or authentic NBI images. The present investigation was retrospective and image-based, and the SAVE-transformed dataset has not been evaluated in a prospectively enrolled patient group.

### 2.2. SAVE

**Figure 1:**
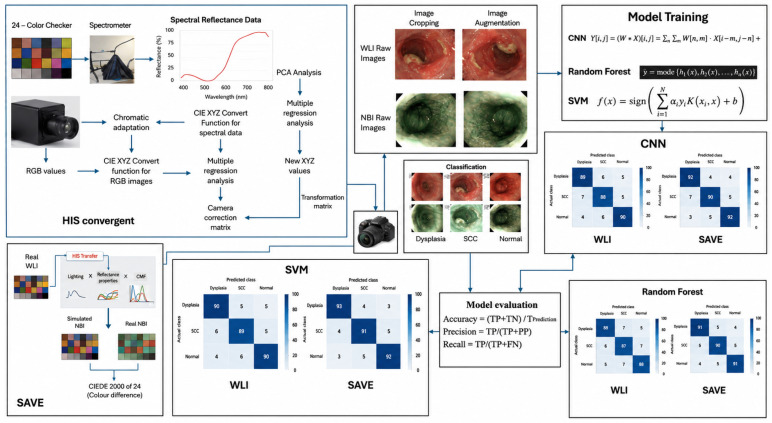
Overall flowchart of the SAVE conversion algorithm.

The SAVE approach has significantly advanced imaging research, paving the way for new color science and imaging technology developments. WLIs were transformed into HSI images to create the SAVE dataset for this investigation. The overall proposed framework for classifying the esophageal tissue, both with and without the SAVE algorithm, is illustrated in Figure 1. The calibration of the WLI image with the spectrometer before transforming it into other colors was crucial. This calibration was possible using a 24-square X-Rite Classic Macbeth color checker (Grand Rapids, MI, USA) that featured a range of natural colors, which included green, red, blue, yellow, cyan, six shades of gray, and magenta. Currently, X-Rite serves as the preferred instrument for color calibration, as the colors captured by the endoscopic camera correspond accurately with those displayed on the X-Rite board. The transformation process involves converting the 24-color patch image into the CIE 1931 XYZ color space. The RGB values in these JPEG photos are converted from 0 to 255 to a smaller range of 0–1. The images are in the sRGB color space. A gamma function converted the scaled sRGB values to linearized RGB values. A translation matrix was then used to transfer the RGB values into the CIE 1931 color space, establishing a numerical correlation between SAVE wavelengths and natural colors. This process requires meticulous calibration for transforming WLIs to SAVE images, a testament to our dedication and attention to detail in this research.

The endoscope can store JPEG images with eight bits because of the sRGB color standard. The RGB values of an sRGB image are then normalized to have values between 0 and 1 before the sRGB values are translated into XYZ. The γ-function gamma-corrects the sRGB values, after which a transformation matrix is utilized on the gamma-corrected sRGB values to yield the corresponding color values in the XYZ color space. The spectrometer used color-matching functions and the light source’s spectrum, S(λ), as procedural steps to convert the reflection spectrum data, ranging from 380 to 780 nm with a resolution of 1 nm, into the XYZ color space. The brightness values were modified to ensure that the brightness level corresponded appropriately to the Y value within the XYZ color system. On the contrary, the acquired reflectance spectrum data were normalized using XYZ, meaning the brightness value ran from 0 to 100. In addition, the reflection data were normalized using this brightness value to produce the luminance ratio k. The camera error conditions—nonlinear response, color filter separation, dark current, and color shift—were added to the variable matrix V. The camera errors were regressed onto V using the correction coefficient matrix C. Utilizing XYZ_Correct_ vs. XYZ_Spectrum_ data, the average RMSE was 0.5355.

(1) [C] = [XYZ_Spectrum_] × pinv([V])

(2) [XYZ_Correct_] = [C] × [V]

In the second research, the data from R_Spectrum_—the spectrum of reflection data measured via the spectrometer for the 24 shades following calibration—was compared to the data from XYZ_Correct_. Following PCA’s identification of R_Spectrum_’s key principal components, multiple regression analysis was employed to create the conversion matrix, M. The selection of V_Color_ of XYZ_Correct_ and score allowed for the completion of multivariate regression analysis because their list of all potential permutations of X, Y, and Z is complete and exhaustive. Also, the S_Spectrum_ application was used to compare the range of the reflection from 24 color blocks with their analog spectra. The average RMSE of each color block was 0.0532 after comparing it with the program. Additionally, it is possible to see the differences in color between S_Spectrum_ and R_Spectrum_. This represented the concluding phase in the development of the VIS-HSI algorithm, designed to precisely replicate the RGB reflection spectrum of the camera.

(3) [M] = [Score] × pinv([V])

(4) [S_Spectrum_]380~780 nm = [EV][M][V_Color_]

The HSI conversion technique was developed for the detection and classification of WLI and EC images to SAVE, utilizing VCE endoscopes and Olympus technology. VCE endoscopes cannot capture SAVE images directly like Olympus endoscopes. The HSI conversion algorithm generated a SAVE image and the Olympus endoscope captured an actual SAVE image. The simulated and authentic SAVE images were evaluated using an average 24-color Macbeth checker. The CIEDE 2000 color difference was calculated between the 24 color blocks, resulting in a minimized average color difference of 2.79. Three components contribute to the disparity in color observed between the actual and simulated SAVE images: the reflection spectrum, the function light spectrum, and the process of color-matching. The light spectrum was standardized using the Cauchy–Lorentz distribution.


(5) $$f(x;x_{0},\gamma )=\frac{1}{\pi \gamma [1+{\left(\frac{x-x_{0}}{\gamma }\right)}^{2}]}=\frac{1}{\pi }[\frac{\gamma }{{\left(x-x_{0}\right)}^{2}+\gamma^{2}}]$$

**Figure 2:**
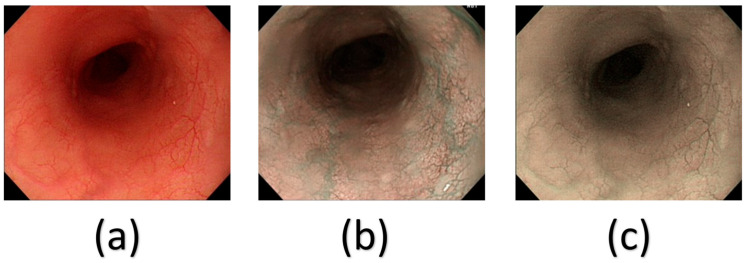
Comparison of the WLI (**a**), a similar original NBI (**b**), and the corresponding SAVE image of the WLI image (**c**).

The VCE representation of SAVE and the corresponding Olympus NBI image underwent calibration using Macbeth 24-color checkers once more. The peak absorption wavelengths of hemoglobin were identified to range from 415 to 540 nm. The Olympus endoscope’s actual NBI image displays shades of brown alongside green and blue, correlating with a wavelength of 650 nm. Consequently, it can be concluded that the NBI videos have experienced slight post-processing enhancements to augment their realism. Consequently, this study includes three additional locations within the wavelength range of 600, 700, and 780 nm, alongside the previously mentioned wavelengths of 415 and 540 nm. Figure 2a shows the traditional WLIs and Figure 2c shows the respective SAVE images in comparison with the original NBI images (Figure 2b).

### 2.3. ML Algorithms

#### 2.3.1. Convolutional Neural Network

Convolutional neural network (CNN) is a deep learning algorithm for object detection, classification, and segmentation [32]. Visual data, like photos and videos, can be analyzed by models like CNN. It takes inspiration from the human visual system, i.e., the human visual cortex, CNN, which has layers that extract simple features first and then build more complex representations [33]. The convolutional layers serve as the fundamental components of the CNN architecture. The layers implement various filters, referred to as kernels, on input images to extract features such as edges, textures, and patterns. The complete operation involves sliding these kernels across the input image and producing feature maps by computing dot products [34]. The formula can calculate the output of a convolutional layer Y:

(6) Y[i,j] = (*W* ∗ *X*)[*i*,*j*] = ∑_m_ ∑_n_ *W*[*m*, *n*] ∙ *X*[*i* − *m*,*j* − *n*] + *b*

In this context, W denotes the filter weights, X signifies the feature map or input image, and b refers to the bias term incorporated into each output. Each layer has multiple times of such data and calculations can have a high memory cost. Hence, to reduce the spatial dimensions, MaxPooling was used, which downsamples the feature map by selecting the maximum within each pooling window [35].

(7) Y[i,j] = max (X[i ∙ s: i ∙ s + f, j ∙ s: j ∙ s + f])

Here, s denotes the stride and f represents the size of the pooling window.

Subsequent to the application of multiple layers, feature maps undergo a flattening process, transforming into a vector that serves as input for the fully connected layers. These layers assist in classification by learning to extract features related to each class from the map. A standard fully connected layer calculates its outputs as z

(8) *z* = *W* ∙ *X* + b

In this context, X represents the input vector derived from the preceding layer, b denotes the bias vector, and W signifies the weight. This study employed a model comprising four convolutional layers. The initial layer contained 32 filters, while the subsequent three layers each incorporated 64 filters, utilizing the ‘ReLU (Rectified Linear Unit)’ activation function. Following each convolutional layer, a MaxPooling layer was implemented to reduce the dimensionality of the input.

The final layer of a CNN is consistently equipped with an activation function. This aspect is significant as it transforms the raw output into a probability distribution, thus enhancing the interpretability of the model’s predictions [36].

(9) $$\mathrm{softmax}{\left(z\right)}_{i}=\frac{e^{z_{i}}}{\sum_{j=1}^{K}e^{z_{j}}}$$

The Adam Optimizer functions to modify all weights and biases within the model. This method is particularly effective, adapting the learning rates of all parameters and ensuring very efficient training [37]. In the CNN model, all input images were scaled to 640 × 640 × 3 pixels. The network had four convolutional blocks. The first convolutional layer contained 32 filters, whereas both the second and third convolutional layers comprised 64 filters each. Each convolutional layer was preceded by a ReLU activation function and succeeded by a MaxPooling layer to diminish spatial dimensionality. The retrieved feature maps were flattened and forwarded to the final classification layer, which had three output classes: normal, dysplasia, and SCC. The output layer employed a softmax activation function for multi-class classification. The model was trained using the Adam Optimizer and the cross-entropy loss function. This CNN model lacked transfer learning and a pre-trained backbone. The CNN architecture was kept very simple, and thus, no extensive hyperparameter optimization was performed; instead, the identical fixed design was utilized for both WLI and SAVE datasets to facilitate a fair comparison of imaging modalities. Grad-CAM analysis was performed on the CNN model to improve interpretability. Grad-CAM heatmaps were generated on the final convolutional layer to pinpoint regions of the picture that significantly influenced the model’s classification decision. The heatmaps were then superimposed on the original endoscopic images to subjectively assess whether the CNN focused on clinically significant mucosal and lesion-related regions rather than extraneous background areas. This explainability study was developed as a visual tool for interpreting the CNN predictions.

#### 2.3.2. Random Forest

Random Forest is a strong ensemble machine learning algorithm that aggregates the predictions of numerous decision trees, thereby enhancing overall accuracy and mitigating the risk of overfitting. In each decision tree, a random subset of data along with various random subsets of features are selected at each split point [38]. This technique is known as bootstrap aggregating or bagging. The output is decided by consolidating the predictions of each tree through majority voting [39].

The formula for prediction in Random Forest is written as:

$$\hat{y}=\mathrm{mode}\{h_{1}(x),h_{2}(x),\ldots .,h_{t}(x),\}$$

Here $\hat{y}$ represents the predicted class, h_t_(x) is the prediction of tth number of trees and t is the total number of trees in the model.

Grid Search with cross-validation is used to perform hyperparameter tuning. It is used to find the optimal combination of hyperparameters that gives minimum error on the validation set. Two parameters are tuned: n_estimators_, the number of trees in the model, and min_samples_leaf, the minimum number of samples in each leaf node [40].

StratifiedKFold cross-validation method is also used for each combination of hyperparameters. In this method, the dataset is split into k equal folds or subsets and each fold serves as the test set once while the other k-1 folds serve as the training set. The accuracy is computed for each of the k folds and their average provides an estimate of the model’s general performance [41].

The formula used for the StratifiedKFold method is:

(10) $$\mathrm{CV}\;\mathrm{Score}=\frac{1}{k}\sum_{i=1}^{k}{\mathrm{Accuracy}}_{i}$$

Here, *Accuracyi* is the accuracy obtained by the ith fold.

#### 2.3.3. Support Vector Machine

Features derived from the input images are utilized in the Support Vector Machine (SVM) for the classification of images into various categories. The algorithm generates a collection of hyperplanes within a high-dimensional space to facilitate the separation of classes. The SVM finds the hyperplane with the maximum distance between the hyperplane and the nearest data points from each class [42]. This distance is known as the support vector.

The decision function for the SVM classifier is:

(11) $$f(x)=\mathrm{sign}\left(\sum_{i=1}^{N}\alpha_{i}y_{i}K(x_{i},x)+b\right)$$

Here, *αi* represents Lagrange Multipliers, *yi* are class labels, *K*(*xi*, *x*) is the kernel function, and *b* is the bias term.

The kernel function, by default, is the radial basis function (RBF) [43]. It is defined as:

(12) *K*(*xi*, *x*) = *exp*(−*γ*||*x_i_* − *x*||^2^)

where *γ* determines the width of the Gaussian function.

Hyperparameter tuning and cross-validation are performed in the SVM, too. The different parameter sets in this model include the regularization parameter C, the kernel coefficient [44] *γ*, and the kernel type. Achieving a low testing error and a low training error is controlled by the regularization parameter C, while the decision boundary’s complexity is influenced by *γ* [45].

#### 2.3.4. Evaluation Indices

The assessment metrics employed in this study include accuracy, precision, recall, and F1 score. Accuracy quantifies the ratio of correct predictions to the total number of predictions generated by the model [46]. Accuracy serves as a general metric for evaluating model performance; however, it may present a misleading representation in the context of imbalanced datasets. This is due to the potential of the model to exhibit strong performance on majority classes while demonstrating suboptimal results in minority classes [47].

(13) Accuracy = (True Positives + True Negatives)/Total Predictions

Precision is the measure of all the positives predicted by the model. That means it indicates the accurate favorable prediction proportions among all optimistic predictions. This becomes particularly important when false positives come with high costs, like medical diagnoses [48].

(14) Precision = True Positives/(True Positives + False Positives

Recall pertains to true positive instances that are accurately identified by the model. This is crucial in instances where positive cases may be overlooked, particularly in scenarios where disease screening incurs significant costs [49].

(15) Recall = True Positives/(True Positives + False Negatives)

The F1 score represents a harmonic mean of precision and recall, serving as a critical metric for evaluating the performance of classification models. The approach effectively addresses the trade-off between false positives and false negatives, particularly in scenarios where class distribution is imbalanced [48].

## 3. Experiment Results

**Figure 3:**
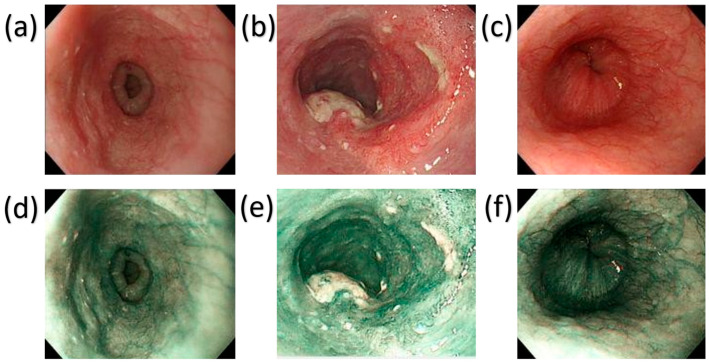
WLIs and corresponding SAVE images. (**a**–**c**) shows dysplasia, SCC, and normal images respectively while (**d**–**f**) shows the corresponding SAVE images.

The training and testing of the models were performed using data that was preprocessed and loaded from the directory. This was done with the help of TensorFlow (https://www.tensorflow.org/, accessed on 15 May 2026). The basic parameters were set before importing the input images, like the size of the image and the number of color channels (three for WLIs). The input data was then finally loaded from the home directory using TensorFlow. Each input image was standardized to 640 × 640 and the dataset was shuffled while importing to improve model training performance. Once all the images were imported, they were split into training, validation, and testing datasets in a ratio of 85:05:10 for CNN and training and testing datasets of 85:15 for all other models. Since the pictures were shuffled while loading the training and testing dataset, they differed every time the code was run. Pre-training augmentation has already been performed on some images using the Albumentations library of Python 3.7, which includes horizontal flips, vertical flips, 90-degree rotation, and 45-degree-range rotation, each with a ratio of 0.5. This process enhanced the models’ robustness by increasing image representations’ diverseness. The evaluation metrics, namely precision, accuracy, F1 score, and recall played an important role in evaluating the performance of the models. Table 2 presents the summary of the results of each model and for each of the evaluation indices for two types of images, WLI and SAVE. Visual examples of the WLI images and their corresponding SAVE transformations for Dysplasia, SCC, and Normal classes are provided in Figure 3. The study showed that CNN’s use of SAVE images showed excellent results—100% in all the evaluation indices. That means a complete model that can predict all the photos from the testing dataset with complete accuracy was made using SAVE images. In contrast, the model that used WLIs had an accuracy of 93%. It showed a very high recall of 96% for the normal class and the same with precision of 96% for SCC. CNN also has the best performance among all other models used in this study because it has four convolutional layers with a ‘ReLU’ activation function, each combined with a MaxPooling layer that selects the maximum within a pooling window of 2 × 2 that downsamples the feature map, reducing the computational cost.

The next highest jump in performance can be seen in the results of Random Forest without hyperparameter tuning. The accuracy went from 91% in WLIs to 96% in SAVE images as shown in Table 2. The capability of detecting SCC also saw a significant increase, with the precision, recall, and F1 score for WLIs being 89%, 94%, and 93%, respectively, and rising to 97%, 98%, and 98%, respectively, for SAVE images. Other classes also experienced increased accuracy in SAVE compared to WLI. For instance, the precision for dysplasia went from 91% in WLI to 96% in SAVE. Similarly, the recall for the normal class went from 87% in WLI to 98% in SAVE. Considering the Random Forest with hyperparameter tuning, all evaluation indices increased in SAVE compared to WLI by 2 to 3%, except the recall of the normal class, which jumped around 8% from 85% to 93%. Now, comparing the two Random Forests, with and without hyperparameter tuning, in this study, there is a decrease in accuracy in the one with hyperparameter tuning as compared to the one without it. One of the reasons might be overfitting, which is quite common when hyperparameter tuning is used. Even then, although smaller, the relative jumps in the evaluation metrics between WLI and SAVE remain similar in both models. SVM had the lowest results compared to all other models, even though it showed a 5% increase in accuracy from WLI to SAVE. The recall of SCC saw a fall, from 88% in WLI to 83% in SAVE, as well as the precision of dysplasia, which went to 81% in SAVE from 84% in WLI. However, the overall accuracy and F1 score increased by 3% in dysplasia, 4% in SCC, and 7% in the normal class.

**Figure 4:**
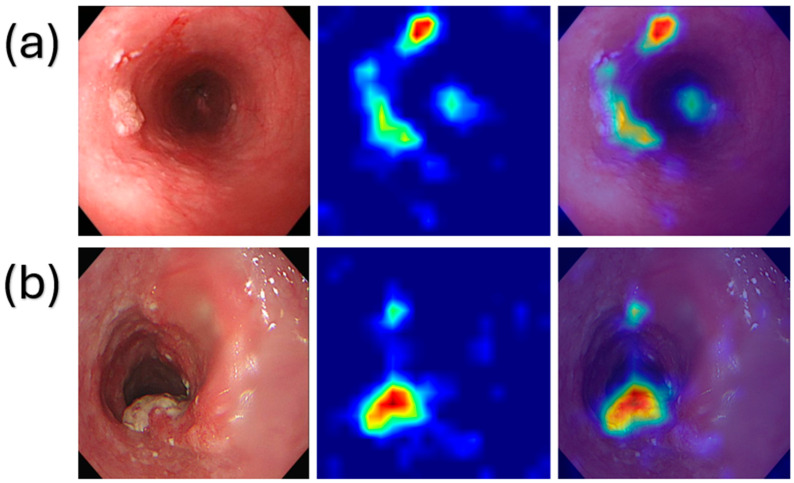
Grad-CAM visualization of (**a**) dysplasia and (**b**) SCC.

The Grad-CAM visualization indicated that the CNN model mostly concentrated on mucosal regions exhibiting discernible pathological changes, including aberrant mucosal texture, discoloration, uneven surface patterns, and areas associated with lesions. The activation regions depicted in Figure 4 were mostly concentrated over clinically significant esophageal mucosal patches rather than the periphery black borders or extraneous backdrop. The results demonstrate that the CNN predictions were influenced by diagnostically relevant picture regions. Grad-CAM, however, does not provide a quantitative elucidation of model attention and cannot be construed as a quantitative clinical validation. Further assessment by experienced endoscopists and possible validation will be required to ascertain whether the discovered regions consistently correlate with clinically and histopathologically relevant lesions.

Further statistical analysis was done based on the final confusion matrices of the held-out test datasets. It was reported to be accurate with 95% confidence intervals calculated using the Wilson score method, and other performance indices, including macro-averaged F1 score, balanced accuracy, and Cohen kappa, calculated. In the CNN model, SAVE images showed a statistically significant improvement over WLIs, with accuracy increasing from 93.2% to 100.0% (*p* = 0.0004). The corresponding macro-F1 score, balanced accuracy, and Cohen kappa also increased correspondingly to WLI 93.2, 93.6, and 0.898 to SAVE 100.0, 100.0, and 1.000, respectively. In the case of Random Forest with no hyperparameter tuning, the accuracy increased by 4.6% [95% CI: 4.1 to 5.2%] when using WLIs as opposed to 5.6% [95% CI: 5.1 to 6.1%] when using SAVE images, though this difference did not reach statistical significance in the aggregate count comparison [*p* = 0.120]. The macro-F1 score, balanced accuracy, and Cohen’s kappa improved from 90.3%, 90.1%, and 0.857 to 95.6%, 95.7%, and 0.934, respectively. For Random Forest with hyperparameter tuning, accuracy increased from 90.0% [95% CI: 84.4–93.8%] for WLI to 93.1% [95% CI: 88.1–96.1%] for SAVE [*p* = 0.422]. Similarly, the SVM model showed an improvement in accuracy from 79.4% [95% CI: 72.5–84.9%] for WLI to 83.8% [95% CI: 77.3–88.7%] for SAVE [*p* = 0.387], with macro-F1 score, balanced accuracy, and Cohen’s kappa increasing from 79.3%, 79.1%, and 0.689 to 83.8%, 83.7%, and 0.756, respectively. All these supplementary analyses confirm the pattern that SAVE-transformed images enhanced the performance of classification in all models assessed. But in all models, other than the CNN model, the improvements were not statistically significant using aggregate confusion-matrix-based tests. The main objective of our work, which was to enhance the diagnosis of early-stage EC by utilizing contemporary imaging and AI technologies, is closely aligned with notable improvements in accuracy, especially in detecting SCC and dysplasia. Since it directly influenced the likelihood of lowering death rates associated with the detection of advanced-stage cancer, ensuring this alignment was essential. The ability of HSI to provide more detailed information on tissue properties and to capture a larger spectrum of light significantly improved the model’s ability to distinguish and accurately identify the characteristics of SCC. HSI may be helpful for applications that require great diagnostic accuracy in recognizing and characterizing complex medical disorders.

## 4. Discussion

ESCC is rarely diagnosed in time due to its asymptomatic characteristics in its early stage and hence, its early detection can significantly enhance the survival rate. Here, the evaluation indices showed promising results for not just dysplasia but also suggested that images of SAVE show greater accuracy in the classification of SCC, dysplasia, and regular classes than traditional WLIs. This study showcased the capabilities of algorithms capable of converting WLIs to SAVE images, which can further enhance the classification. Though accuracy and other evaluation metrics show significant results, the dataset size was smaller than conventional datasets used in most studies, with only around 1074 images. Hence, further validation is expected once the dataset size is increased to include more variety in the current classes. As for the models used in this study, the results for SVM were poorer than for the other models because SVM is more straightforward and less complex. Also, generally, SVMs require hyperparameter tuning to provide the best output, which is computationally expensive on large datasets. As for the Random Forest, it provided poorer results when hyperparameter tuning was used than when it comes to those without hyperparameter tuning. It is possible that the model was trained too closely to the training data that it overfitted, thus hurting the generalization of new unseen data. CNN was the most complex model among the three, with four layers, excluding the final layer. CNNs are also better equipped to handle extensive data since they are nonlinear and can easily capture complex patterns. All models used in this study were basic; they had no complex parts. Better and more complex models can be used in future studies to enhance the results. The present findings should be considered in relation to prior AI-driven investigations utilizing RGB/WLI, NBI, or hardware-based hyperspectral imaging for the identification of esophageal cancer. Despite past RGB-based deep learning experiments exhibiting commendable diagnostic efficacy, it remains ambiguous how to directly juxtapose the current SAVE-based methodology with the former RGB-based technique. SAVE is a distinctive technique capable of computationally converting regular RGB/WLIs into hyperspectral-like, NBI-enhanced images without requiring specialized HSI hardware. Consequently, a direct comparison of SAVE with just RGB-based methods or hardware-dependent HSI systems may not accurately represent its methodological innovation. The improved performance noted in this work suggests that computational spectrum augmentation can offer supplementary diagnostic information relative to standard WLI; however, bigger external studies are necessary for adequate benchmarking and validation. The sample size was quite small, consisting of 762 initial WLI photos supplemented to 1074 images, which may restrict the generalizability of the findings. Secondly, all images were obtained from a single institution, hence the data may not comprehensively reflect the variations in imaging circumstances, endoscopic systems, patient demographics, and disease presentations among different clinical centers. Third, although data augmentation was utilized to improve model robustness, augmented images may not fully replace independent clinical data. Future research must ensure patient-level separation among training, validation, and testing datasets to mitigate the risk of data leakage. The current categorization task encompasses three categories (normal, dysplasia, and SCC), whereas actual clinical diagnosis may include other categories such as inflammation, hemorrhage, Barrett’s esophagus, and other benign or malignant lesions. Finally, the SAVE technique has not yet been prospectively validated in standard clinical practice. Therefore, more multi-center studies incorporating external validation, patient-level analysis, and prospective clinical evaluation are necessary to confirm the diagnostic efficacy and robustness of the proposed SAVE-based classification framework.

## 5. Conclusions

Esophageal cancer has one of the highest mortality rates among all cancers. Hence, if not detected early, the chances of that patient surviving can be meager. This study aims to enhance EC detection by combining SAVE with machine learning models like Random Forests, CNN, and SVM. The approach outperformed 16 traditional imaging techniques regarding accuracy and other evaluation metrics. The SAVE technique converted regular WLIs into SAVE images to enhance the model’s classification ability. After that, both WLIs and SAVE images were evaluated through multiple models using error metrics such as accuracy, precision, recall, and F1 score. It was found that using SAVE images proved to be more accurate than WLIs for classifying different classes of EC. Every model increased accuracy by at least 3% in SAVE images compared to traditional WLIs. This study, hence, filled the voids in the current medical diagnosis system by offering a more efficient method that can be used for early diagnosis of EC. This approach may provide a useful foundation for future development of computer-aided esophageal cancer screening tools. However, its clinical utility, effect on early detection, patient outcomes, and cost-effectiveness must be confirmed through larger prospective and multi-center studies. The research aimed to improve the existing detection methods and provide the foundation for further study in advancing medical imaging.

## Figures and Tables

**Table 1:** Dataset used in the study before and after augmentation.

Class Name	Images Before Augmentation	Images Augmented	Images After Augmentation
Normal	243	93	336
Dysplasia	278	128	406
SCC	241	91	332
Total	762	312	1074

**Table 2:** Results of RF with and without hyperparameter tuning, CNN, and SVM in terms of precision, recall, F1 score, and accuracy.

Random Forest (Without Hyperparameter Tuning)					
WLI	Class	Precision	Recall	F1 Score	Accuracy
	Normal	91%	87%	89%	91%
	Dysplasia	91%	89%	89%	
	SCC	89%	94%	93%	
SAVE	Class	Precision	Recall	F1 Score	Accuracy
	Normal	93%	98%	96%	96%
	Dysplasia	96%	91%	94%	
	SCC	97%	98%	98%	
Random Forest (With Hyperparameter Tuning)					
WLI	Class	Precision	Recall	F1 Score	Accuracy
	Normal	91%	85%	88%	90%
	Dysplasia	91%	89%	90%	
	SCC	89%	94%	91%	
SAVE	Class	Precision	Recall	F1 Score	Accuracy
	Normal	93%	93%	93%	93%
	Dysplasia	94%	91%	93%	
	SCC	92%	95%	93%	
CNN					
WLI	Class	Precision	Recall	F1 Score	Accuracy
	Normal	93%	96%	95%	93%
	Dysplasia	90%	94%	92%	
	SCC	96%	90%	93%	
SAVE	Class	Precision	Recall	F1 Score	Accuracy
	Normal	100%	100%	100%	100%
	Dysplasia	100%	100%	100%	
	SCC	100%	100%	100%	
SVM					
WLI	Class	Precision	Recall	F1 Score	Accuracy
	Normal	83%	73%	78%	79%
	Dysplasia	84%	77%	80%	
	SCC	73%	88%	80%	
SAVE	Class	Precision	Recall	F1 Score	Accuracy
	Normal	86%	84%	85%	84%
	Dysplasia	81%	84%	83%	
	SCC	85%	83%	84%	

## Data Availability

The data presented in this study are available on request from the corresponding author. The data are not publicly available due to strict privacy and ethical restrictions mandated by the Institutional Review Board of Ditmanson Medical Foundation Chia-Yi Christian Hospital (Approval No. IRB2025099).
